# Supplementary material for: Alkaloid Profile Characterisation and Bioactivity Evaluation of Bolivian Hippeastrum Species (Amaryllidaceae) as Cholinesterase Inhibitors
Source: Life (Basel). 2025 Apr 29;15(5):719. doi: 10.3390/life15050719 (PMC12113584; doi:10.3390/life15050719)
Supplement: Supplementary file 1 [file life-15-00719-s001.zip › life-3530949-supplementary.pdf]

Table S1. Alkaloid profile and individual alkaloid concentration values of Bolivian *Hippeastrum* species. Values are expressed in µg Gal/100 mg of dry weight (DW).

Alkaloids	RI	RT	[M$^+$]	H1	H2	H3	H4	H5	H6	H7	H8	H9	H10	H11	H12	H13	H14	H15	H16	H17	H18	H19	H20	H21	H22	H23	H24	H25	H26	H27
**Lycorine-type (total)**				**0**	**80.8**	**0**	**0**	**0**	**1.1**	**13.8**	**7.6**	**49.4**	**0**	**0**	**0**	**0**	**0**	**33.2**	**8.1**	**0**	**0**	**8.5**	**0**	**0**	**0**	**0**	**0**	**0**	**0**	**59.2**
Anhydrolycorine (1)	2818.0	24.6375	250									12.5																		9.6
Assoanine (2)	2909.0	25.7095	266		7.1																									
Dihydrolycorine (3)	2745.0	23.7785	288								7.6							16.5												
Galanthine (4)	3022.4	27.0459	242		39.1				1.1										4.2											
Lycorine (5)	3138.3	28.4106	226		7.3					13.9		25.2						16.7				4.4								39.8
Tortuosine (6)	3212.8	29.2889	296		20.6																									
11,12-Dehydroanhydrolycorine (7)	2948.8	29.1784	248		6.8							11.7							3.9			4.1								9.8
**Homolycorine-type (total)**				**5.6**	**0.0**	**28.9**	**37.6**	**44.6**	**0.0**	**11.9**	**60.4**	**0.0**	**0.0**	**0.0**	**15.1**	**17.4**	**18.6**	**0.0**	**13.2**	**0.0**	**5.4**	**8.1**	**12.9**	**0.0**	**0.0**	**5.7**	**0.0**	**22.2**	**0.0**	**0.0**
Candimine (8)	3305.2	30.3770	125			15.0		15.0																						
Cliviasine (9)	3181.0	28.9140	96																		1.4									
Hippeastrine (10)	3269.9	29.9614	125	1.8			12.3	14.8													2.5		4.8							
Homolycorine (11)	2624.8	22.3619	109								36.4					7.4	8.4		9.2			4.0	4.0			5.7				
Nerinine (12)	3177.9	28.8780	109	1.9		13.9					24.0					10.1	10.2				1.5	4.1	4.0					14.5		
8-O-Demethylhomolycorin*e (13)*	2739.7	27.0306	109	1.9						11.9					8.6				4.0											
2-Hydroxyhomolycorine (14)	3329.0	30.6576	109				13.3																					7.7		
2-Methoxy-8-O-demethylhomolycorine (15)	3021.1	27.0308	109				12.1	14.8							6.5															
**Galanthindole-type (total)**				**2.0**	**0.0**	**0.0**	**0.0**	**0.0**	**0.0**	**0.0**	**7.8**	**0.0**	**0.0**	**0.0**	**6.5**	**7.3**	**0.0**	**0.0**	**0.0**	**0.0**	**1.6**	**0.0**	**0.0**	**0.0**	**0.0**	**0.0**	**0.0**	**16.0**	**0.0**	**0.0**
Galanthindole (16)	2553.9	24.1963	281	2.0							7.8				6.5	7.3					1.6							16.0		
**Crinine/Haemanthamine-type (total)**				**3.5**	**13.8**	**0.0**	**0.0**	**0.0**	**0.0**	**14.5**	**7.8**	**26.0**	**0.0**	**8.7**	**0.0**	**23.6**	**0.0**	**0.0**	**4.0**	**0.0**	**0.0**	**0.0**	**0.0**	**0.0**	**0.0**	**0.0**	**0.0**	**0.0**	**45.9**	**9.9**
Maritidine (17)	2566.1	21.6712	287		6.8											8.1														
O-Methylmaritidine (18)	2761.6	23.9741	301													15.5														
Vittatine/crinine (19)	2769.7	24.0685	271	1.8								12.0		8.7															7.4	
8-*O*-Demethylmaritidine (20)	2810.6	24.5511	273							14.5		14.0																	8.3	
Crinane-3-one (21)	2852.4	27.5250	271/181																										7.3	
Crinamine (22)	2945.0	26.1345	272																4.0											
Hippeastidine (23)	2947.4	26.1626	319								7.8																			
Haemanthamine (24)	3008.2	26.8789	272		7.0																								22.9	
Flexinine (25)	3042.0	27.2765	258																											9.9
Undulatine (26)	3123.8	28.2400	331	1.7																										
**Narciclasine-type (total)**				**1.7**	**0.0**	**0.0**	**0.0**	**14.8**	**0.0**	**0.0**	**7.6**	**0.0**	**16.1**	**0.0**	**0.0**	**7.3**	**0.0**	**0.0**	**0.0**	**15.9**	**1.4**	**0.0**	**0.0**	**0.0**	**0.0**	**5.6**	**0.0**	**7.9**	**7.4**	**0.0**
Trisphaeridine (27)	2346.1	20.9836	223	1.7				14.8			7.6		16.1			7.3				15.9	1.4					5.6		7.9	7.4	
**Pretazettine-type (total)**				**18.6**	**0.0**	**0.0**	**12.2**	**30.7**	**1.0**	**11.9**	**0.0**	**11.1**	**17.1**	**0.0**	**18.3**	**17.4**	**16.4**	**0.0**	**0.0**	**54.5**	**3.1**	**0.0**	**0.0**	**0.0**	**0.0**	**14.7**	**0.0**	**35.7**	**0.0**	**0.0**
Isotazettino (28)	3077.1	27.6901	247																									7.7		
3-*O*-Demethyltazettine (29)	3049.9	27.3696	247							11.9																				
3-*epi*-Macronine) (30)	3186.6	28.9797	245	1.8				14.8								7.6					1.4					5.8		7.7		
Tazettine (31)	2991.6	26.6831	247	13.0			12.2	15.9	1.0			11.1	17.1		6.6	9.8	8.3			15.9	1.7					8.8		12.5		
O-Methyltazettine (32)	2935.3	26.0200	261	3.8													8.1			38.6								7.8		
5-Methoxyhomolycorine (33)	3346.8	30.8677	139												11.7															
**Montanine-type (total)**				**0.0**	**8.1**	**13.7**	**0.0**	**0.0**	**0.0**	**27.9**	**11.2**	**0.0**	**0.0**	**0.0**	**0.0**	**0.0**	**0.0**	**16.8**	**0.0**	**0.0**	**0.0**	**4.1**	**0.0**	**0.0**	**0.6**	**0.0**	**28.0**	**0.0**	**0.0**	**0.0**
Pancracine (34)	3027.5	27.1062	287							16.1																	4.6			
Pancratinine C (35)	2893.0	25.5216	287							11.8								16.8									4.3			
2-*O*-Methylpancracine (36)	2967.6	26.4007	301		8.1	13.7					11.2											4.1			0.6		19.1			
**Galanthamine-type (total)**				**0.0**	**13.5**	**0.0**	**12.4**	**14.9**	**7.5**	**38.6**	**23.8**	**0.0**	**0.0**	**8.7**	**0.0**	**0.0**	**0.0**	**36.5**	**0.0**	**0.0**	**0.0**	**0.0**	**0.0**	**0.0**	**0.0**	**0.0**	**4.3**	**0.0**	**0.0**	**33.0**
Sanguinine (37)	2666.4	22.8524	273						1.1																					
Galanthamine (38)	2406.0	22.5833	286															20.0												14.5
Narwedine (39)	2746.7	23.7978	284						1.3		7.6							16.5												9.2
3-*O*-Acetylgalanthamine (40)	2824.7	24.7172	270						1.1																					
Lycoramine (41)	2699.5	23.2424	288		6.7			14.9		23.8	7.8																			9.3
Lycoraminone (42)	2731.4	23.6183	286								8.3																			
Galanthamine-*N*-oxide (43)	2638.4	22.5226	286				12.4																							
Epigalanthamine (44)	2670.0	22.8942	287		6.7				4.1					8.7													4.3			
Norlycoramine (45)	2736.6	23.6786	274							14.8																				
**Ismine-type (total)**				**1.8**	**6.7**	**0.0**	**12.1**	**15.0**	**0.0**	**11.8**	**9.3**	**11.1**	**0.0**	**0.0**	**6.5**	**8.5**	**8.1**	**0.0**	**3.9**	**0.0**	**1.6**	**0.0**	**0.0**	**0.0**	**0.6**	**6.3**	**0.0**	**12.9**	**0.0**	**0.0**
Ismine (46)	2497.4	20.6170	238	1.8	6.7		12.1	15.0		11.8	9.3	11.1			6.5	8.5	8.1		3.9		1.6				0.6	6.3		12.9		
**OtherType of alkaloids-total**				**1.8**	**0.0**	**0.0**	**0.0**	**0.0**	**0.0**	**0.0**	**0.0**	**0.0**	**0.0**	**0.0**	**0.0**	**7.3**	**0.0**	**0.0**	**0.0**	**0.0**	**2.8**	**0.0**	**0.0**	**0.0**	**0.0**	**0.0**	**0.0**	**15.3**	**7.2**	**0.0**
5,6-Dihydrolycorine (47)	2384.9	21.6068	238	1.8												7.3					1.4							7.7		
Demethylismine (48)	2473.5	20.5799	224																		1.4							7.6	7.2	
